# Health care needs assessment among adolescents in correctional institutions in Zambia: an ethical analysis

**DOI:** 10.1186/s12913-017-2532-5

**Published:** 2017-08-22

**Authors:** Maureen Kumwenda, Selestine Nzala, Joseph M. Zulu

**Affiliations:** 1 School of Public Health, Department of Health Promotion and Education, University of Zambia, P.O. Box 50110, Lusaka, Zambia; 2 Minstry of Health, District, Lusaka, Zambia; 3 Departments of Public Health, Section of Health Policy and Management, School of Medicine, University of Zambia, P.O. Box 50110, Lusaka, Zambia

**Keywords:** Health needs assessment, Autonomy, justice, beneficence, Correctional facilities

## Abstract

**Background:**

While health care needs assessments have been conducted among juveniles or adolescents by researchers in developed countries, assessments using an ethics framework particularly in developing countries are lacking. We analysed the health care needs among adolescents at the Nakambala Correctional Institution in Zambia, using the Beauchamp and Childress ethics framework. The ethics approach facilitated analysis of moral injustices or dilemmas triggered by health care needs at the individual (adolescent) level.

**Methods:**

The research team utilized 35 in-depth interviews with juveniles, 6 key informant interviews and 2 focus group discussions to collect data. We analysed the data using thematic analysis**.** The use of three sources of data facilitated triangulation of data.

**Results:**

Common health problems included HIV/AIDS, STIs, flu, diarrhoea, rashes, and malaria. Although there are some health promotion strategies at the Nakambala Approved School, the respondents classified the health care system as inadequate. The unfavourable social context which included clouded rooms and lack of adolescent health friendly services unfairly exposed adolescents to several health risks and behaviours thus undermining the ethics principle of social justice. In addition, the limited prioritisation of adolescent centres by the stakeholders and erratic funding also worsened injustices by weakening the health care system. Whereas the inadequate medical and drug supplies, shortage of health workers in the nearby health facilities and weak referral systems excluded the juveniles from enjoying maximum health benefits thus undermining adolescents’ wellbeing or beneficence. Inadequate medical and drug supplies as well as non-availability of adolescent friendly health services at the nearest health facility did not only affect social justice and beneficence ethics principles but also threatened juveniles’ privacy, liberty and confidentiality as well as autonomy with regard to health service utilisation.

**Conclusion:**

Adequately addressing the health needs in correctional institutions may require adopting an ethics framework in conducting health needs assessment. An ethics approach is important because it facilitates understanding of moral dilemmas that arise due to health needs. Furthermore, strategies for addressing health needs related to one ethics principle may have a positive ripple effect over other health needs as the principles are intertwined thus facilitating a comprehensive response to health needs.

## Background

Adolescents in juvenile correctional systems are a high- risk population who in many cases have unmet physical, developmental and mental health needs [1]. Therefore, needs assessments are important because they provide a possibility of gathering information required to bring about positive interventions to the affected adolescents [2]. The overall aim of health needs assessments is to provide information to guide planning, negotiating and improving of health services [3]. Healthcare needs include health education, disease prevention, diagnosis, treatment, rehabilitation and terminal care [4]. According to Majeed [5], needs assessment means different things according to who uses the term, when and where. Health care needs assessments are important because the assessments provide baseline data that can guide the design of interventions that are appropriate and relevant to the target group.

According to Katherine [6], over 1 million children worldwide are detained by law enforcement bodies. Juvenile detainees have been identified as a group that participates in high risk behaviors including substance abuse, early sexual activity, violence, weapon use, murder and gang involvement. This group also has a high prevalence of medical conditions including seizure disorders, respiratory diseases, nutritional deficiencies, orthopedic skin and dental problems [7]. The American Academy of Pediatrics [1] also adds that many problems afflict detained youth and include communicable diseases, especially sexually transmissible infections, hepatitis and tuberculosis (T.B). While data on the prevalence of child incarceration are uneven and sometimes unavailable throughout Africa, the percentage of the prison population comprising of child detainees generally ranges from 0.5% to 2.5%. Namibia where juveniles represent 5.5% of prisoners has the highest reported rate. In April 2010, Zambia’s prisons held 414 juvenile inmates (a category under Zambia’s law encompassing inmates aged 8 to 18), representing 2.5% of all Zambian inmates [6].

Zambia has 3 juvenile correctional institutions namely the Nakambala Approved School in Mazabuka district (Southern Province), Insakwe Approved School in Ndola (Copperbelt Province) and Katombora Reformatory School in Kazungula District (Southern Province). The Schools provide care, protection, rehabilitation, other social support services such as provision of shelter, clothes, food, counselling as well as reintegration of children back into communities. The aim of the institutions is to provide every child a healthy and secure living for them to actively participate in the routine development and rehabilitation. Effective health care services at any institution are key in determining adolescents’ health and meeting the overall institution’s objectives of rehabilitation and reintegration into the community [8].

Zambia has a total of 86 prisons throughout the country, though one of these facilities is exclusively dedicated to juveniles. In some instances, juveniles who are in these facilities are incarcerated with the adult population at other facilities countrywide [6, 9]. Katherine [6] between September 2009 and February 2010 interviewed 246 prisoners as part of study examining human rights conditioning and HIV and tuberculosis prevention, treatment and care in prisons in Zambia. The findings of this study underscore the need for addressing both the criminal justice failures that lead to juveniles being incarcerated for extended periods under deplorable conditions and the particular ways in which juveniles’ treatment in adult facilities expose them to sexual and physical abuse as well as diseases.

Whilst juveniles benefit from some form of social services in correctional institutions, corresponding data or information on health care services in these facilities is not documented [8, 9]. However, at the Nakambala Approved School, there is evidence of serious diseases within the institutions, such as syphilis, gonorrhea, and STIs. Meanwhile, there is no health facility at the institution. Another juvenile institution, Insakwe Approved School in Ndola which had a total number of 89 female juveniles has also been affected by diseases [6, 10, 11].

In the past 10 years, the Nakambala Approved School had a memorandum of understanding with the Ndeke Clinic in Mazabuka district and Mazabuka General Hospital where the Ministry of Health designated a clinical officer and a nurse to periodically undertake clinical assessments on juveniles once a week. But such services are no longer offered at the institution [6, 10, 12].

While health care needs assessments have been conducted by researchers among juveniles especially in developed countries, assessments using an ethics framework particularly in low and middle income countries are lacking. We analysed the health care needs among juveniles at Nakambala Correctional Institution in Zambia, using the Beauchamp and Childress ethics framework. The framework has the following main ethics principles: autonomy, beneficence and justice. Autonomy refers to privacy, self-determination and liberty [13]. Beneficence is about reducing or preventing harms, risks and maximizing benefit over harm. Justice involves distributing burdens and harms fairly (distributive justice), as well as ensuring public participation including those affected, building and maintaining trust or procedural justice [13, 14].

## Methods

The COnsolidated criteria for REporting Qualitative research (COREQ) guidelines [15] guided the development of the methodology section. In line with the COREQ guidelines, the key components of the methodology section included research team, study design and analysis.

### The research team and process

Our research team comprised three researchers with postgraduate training in public health and qualitative research. Two researchers collected the data while the third researcher conducted quality check-ups or verification on the data. The involvement of more than one researcher in data collection helped reduce bias in data collection and reporting. The research process was coordinated by the first author. Prior to the study, permission was sought from the Ministry of Community Development, Mother and Child Health to have access to the Nakambala Training School.

### Study design

We adopted a case study method to explore the situation of health needs among juveniles. A case study design is used when the researcher intends to describe the phenomena under study in detail [16]. The study used qualitative method of data collection. Key informant interviews, focus group discussions and in-depth interviews were used to collect data. Questions covered the six building blocks for health system: *service delivery such as access and barriers to health services; human resources for health such as availability of human resources; governance; medical supplies; health information as well as finance. We integrated an ethics approach within this health systems framework. This integration helped in narrowing down from overall systems focus to moral injustices or dilemmas that health care needs trigger at individual (adolescent/juvenile) level.*

### Study setting

The study was carried out at the Nakambala Approved School in Mazabuka district located in the Southern Province of Zambia. The Nakambala Approved School is about 2.5 km from the main post office in Mazabuka district. This place was purposively selected to enable the researcher get the practical view of health care provision to juveniles as the institution had more juvenile offenders aged between 13 and 19 years compared to other institutions in Zambia.

### Sample size and sampling process

There were two sets of samples in the study. The first one comprised 35 male juveniles, the total number of juveniles/adolescents that were present at the correctional institution. The process of recruiting the adolescents started by informing them about the study as a group. The second step was seeking assent from adolescents. The assent process was conducted with adolescents on an individual basis. All the adolescents that were approached agreed to take part in the interviews.

The second sample comprised 6 key informants who were purposively selected because they were responsible for health service delivery at the correctional institution. The key informants were recruited from their offices after informing them about the study.

### Data collection techniques

#### Review of documents

Relevant documents such as annual reports, quarterly reports, and residential case statistical form reports among others were obtained and reviewed from the  Nakambala Training School and the Department of Social Welfare. We reviewed all the pages in the documents. This was done to enable the researcher gain insight into the operations of officers responsible for health care delivery in correctional institutions [9].

#### In - depth interviews

In-depth semi structured interviews were conducted with the 35 adolescents. The interviews provided an opportunity for researchers to probe and gain deeper insights on health needs and ethical dilemmas. Interviews were conducted in a room, and only one researcher and the adolescent were present during the interview to enhance privacy and confidentiality. Each interview lasted for about 30 min. Below are some of the main questions that were asked during the interviews.

- What are the common health care problems at the institution?
- What are the measures in place for addressing the health care needs?
- How adequate are the measures for addressing health needs?
- How adequate is the referral system?
- How do the health care needs affect the juveniles/adolescents’ autonomy?
- What kind of injustices are triggered by the health care needs among the juveniles/adolescents?
- What specific moral dilemmas arise due to health needs?
- How can the health care needs be addressed?
- In what way, can the problems related to autonomy, beneficence and injustices be addressed among adolescents?

#### Key informant interviews

Six (6) semi structured interviews were conducted with the key informants to gather information on health care provision and each interview lasted for 40 min. A sample was drawn from members of staff from the Nakambala Approved School and other stakeholders from the Department of Social Welfare, as well as DAPP in Mazabuka district. Key informant interviews complemented the data provided by juveniles by going beyond the immediate needs to also focus more on strategic approaches for addressing the health care needs.

#### Focus group discussions

Two (2) focus group discussions involving six (6) members in each group were conducted with twelve juveniles and two key informants. Each focus group discussions lasted for about an hour. The researcher guided the discussions using a discussion guide to ensure effective participation from the juveniles.

### Data analysis

All the interviews were recorded and transcribed verbatim. Once transcribed, the three authors read through the data to develop a good sense of the data set. NVivo (version 10, QSR International) was used to organize and manage the data. A single master code list and definitions were iteratively developed by the researchers. The transcribed data were analyzed using thematic analysis [17]. Thematic analysis involved grouping the data into codes and then grouping the codes  that shared similar meaning into categories. Once developed, the researchers discussed the categories and finally grouped them into themes (Table 1). Thematic analysis was used because it allowed for in-depth exploration of issues contained in the data by means of identifying and matching themes that were similar in the process of analysing data.

**Table 1:** Themes and categories

Theme	Category
The ethical principle of beneficence	- Promoting beneficence through implementing health promotion and preventive services - The disease burden compromising wellbeing or beneficence - Inadequate health services affecting the realization of the ethical principle of beneficence
The case of limited autonomy	- Lack of adolescent health friendly services - Lack of screening services for juveniles
The ethical principle of social justice	- Inadequate documenting of health care needs and referral processes - Limited stakeholder focus on correctional institutions - Unconducive environment contributing to high disease burden - Erratic funding towards health services

### Ethical considerations

Ethical clearance and approval was sought from the Excellency in Research Ethics and Science (ERES) Converge committee, Reference number; 2014-May-022. During the implementation of the study, informed consent was sought from the Principal of the Institution as the participants were below 20 years old and study objectives were explained to participants in detail. The juveniles who are below 20 years old are protected by the Juveniles Act CAP 53 of the Laws of Zambia. In line with the ACT, the Commissioner for Juvenile Welfare was legally empowered to consent for or on behalf of the children with or without the consent of the parents. In this regard, consent to interview the juveniles was sought from the Commissioner for Juvenile Welfare. To ensure anonymity of the participants’ identity, initials of their names or institutions where they belong and date of their participation in the study were not recorded. Respondents were further informed that their responses would be treated with the highest level of confidentiality. Furthermore, participants were notified that they were free to withdraw from the study at any point. Assent was obtained from the juveniles after they fully understood the study.

## Results

### Socio-demographic characteristics

The 35 participants were between 11 and 19 years of age. Most of them were in the range of 14 and 17 years and were male who were admitted to the institution. Most adolescents or juveniles had not stayed for more than 1 year at the institution. On average the juveniles stayed for 5 months. Ten (10) juveniles stayed for more than 5 months.

### The ethical principle of beneficence

Although there are some health strategies at the Nakambala Approved School, overall the respondents classified such strategies as inadequate. Below we discuss the current health strategies in relation to ethics principles.

#### Promoting beneficence or well-being through implementing health promotion and preventive services

The health promotion and preventive services, for promoting well-being or beneficence- within the correctional institution were awareness talks on health personal hygiene and counselling on STIs with the emphasis on HIV/AIDS and Syphilis. The juveniles attend health education on various diseases. However, these talks were conducted by un qualified personnel who use their experiences to teach. The staff at the institution conducted counselling to the juveniles whenever there was need and request.

*“ I learnt some personal hygiene practices, mainly about proper toilet use and the need to regularly wash hands with soap*”

#### The disease burden compromising wellbeing or beneficence

One of the issues which affected promotion of the wellbeing or beneficence among the juveniles or adolescents was the lack of comprehensive health care needs assessment. The study showed that no comprehensive health care needs assessment had been conducted at the institution and yet there were some health care problems among the juveniles. As shown in Table 2 these diseases included diarrhoea, rashes, malaria and Tuberculosis (T.B).

**Table 2:** Diseases at the Nakambala correctional facility

Ailment	Number of boys affected
Headache	10
Stomach pains/diarrhoea	10
Malaria	9
Flu/sneezing	9
Coughing	4
Pain/itching in the eye	3
Pain/itching in the ear	3
Neck pain	2
Sores on the skin due to poor hygiene	2
Rash	1
Sore in the arm due to a fall	1
Pain in the limbs	1
Sores due blisters	1
Numbness in the limbs	1
Chicken pox	1
Chest pain(hotness)	1
Bleeding of the noise and pain	1
Mental problem	1
Heartache	1
Depression	1
Sore throat	1
Dry throat	1
Mosquito bite	1
Bleeding, headache and neck pain	1

#### Inadequate health services affecting the realization of the ethics principle of beneficence

In addition, inadequate health services at the Nakambla Approved School affected enhancement of the wellbeing of the adolescents. There was no health facility within the Nakambala Approved School where the juveniles could be attended to even for minor ailments, all the illnesses regardless of their severity were referred to nearby clinic. The nearby clinic had inadequate medical and drug supplies as well as shortage of human resources for health which affected provision of treatment and care to the juveniles or adolescents.

Provision of drug supplies to the juveniles was a challenge since juveniles were dependent on the nearest health clinic which sometimes lacked appropriate medicines and supplies for some health conditions. There were times when the health workers would examine and prescribe the right drugs for the juvenile but such drugs were unavailable, thus minimising health benefits. It was also reported that there were no health workers attached to the Nakambala Approved School.

*“There are some drugs the nurses cannot prescribe. It becomes a challenge as there is only one clinical officer”* (Key informant interview).

*“We don’t even have a community health worker attached to the Nakambala Approved School”* ( Key informant interview).

Furthermore, there were also no follow ups on medical treatment of the juveniles by qualified health personnel clearly undermining adolescents’ wellbeing. Some juveniles required close monitoring over their illnesses by qualified personnel from the nearby clinic but this did not take place because the staff from the clinic did not follow the juveniles.

*“Currently we are a bit understaffed. So even the staffing issues are really affecting the kind of health care we are giving to our clients”* ( Key informant interview ).

### The case of limited autonomy

The study showed that the right to autonomy was greatly affected among the adolescents as confidentiality and liberty or freedom of choice to services were undermined. Below, we discuss in detail some issues which affected autonomy or capacity for self-determination.

#### Lack of adolescent friendly services

In addition to inadequate services, the clinic where juveniles or adolescents were referred to had no adolescent friendly services. Lack of such services affected adolescent’s right to privacy and autonomy especially in cases where they had sensitive health issues such as HIV and STIs. Some juveniles were not comfortable mixing with adults in reproductive health centers due to some cultural values and religious beliefs which stigmatize sexuality among adolescents.

*“At the clinic where juveniles are referred, there are no adolescent health services. This is a big challenge as some adolescents are shy to openly talk about their sexual related challenges. Such fears worsen their health”* (Key informant interview).

#### Lack of screening services for juveniles

The facilities for screening the juveniles upon arrival at the correctional institution were not available. The screening examination was done by observation and by asking the juveniles their health history by un qualified staff. The lack of such screening services affected the adolescents or juveniles’ awareness of their health status thus limiting uptake of health seeking behaviours or prevention measures. Lack of screening services also affected development of interventions targeting specific individuals with special needs.

### The ethical principle of social justice

The unconducive social context unjustly or unfairly exposed adolescents or juveniles to several health risks or behaviours thus affecting the ethics principle of social justice. In addition, injustice took the form of limited prioritisation of the juvenile or adolescent centres by the stakeholders. Erratic funding also affected realisation of the ethics principle of social justice or fairness. Below the major issues around the ethics principle of social justice are discussed.

#### Sexual abuse and unprotected casual sex resulting in sexually transmitted infections

Some juveniles had contracted sexually transmitted infections (STIs) from sexual abuse by adults in the remand and prison. In addition, some of the juveniles got infected from the nearby community where they engaged in casual sex with the girls. The lack of information of SRH matters affected the juveniles’ ability to protect themselves from STIs. One of the juveniles interviewed explained this as follows:

*“Since I came here I have seen that some juveniles like going in the Zambia Compound and there one may sleep with a girl and contract some STIs..”* (Juvenile in-depth interview).

#### Inadequate documenting of health care needs and referral processes

Health information management systems are necessary in residential correctional institutions as they would help in documenting the information regarding health needs of juveniles. But this was not the case with the Nakambala Approved School because the study found that there were no health records for the juveniles and nothing had been documented so far. This hindered the referral process and follow ups of the health care challenges or needs among the adolescents.

#### Limited stakeholder focus on correctional institutions

The correctional institutions were over shadowed by the main prisons for adults and as such most of the stakeholders were not even aware about their existence. From those who were aware of the work of correctional institutions, a few stakeholders such as DAPP and the church had come on board to help were they could.

*“I think the Government needs to come in and provide a lot of resources. I have always been talking to our DAPP National Office concerning the child welfare fund that we should include this institution”* (Key informant interview).

#### Unconducive environment contributing to high disease burden

Several environmental factors contributed to the high disease burden. Malaria was prevalent at the Nakambala Approved School because the school was near the largest sugar plantation in Zambia. Because of sugar plants and stagnant water from irrigation activities, the mosquitoes bred and multiplied easily making the area prone to malaria: Whereas the problem of tuberculosis at the Approved School was closely linked to HIV/AIDS cases, low body immunity was due to factors like poor diet, general poor health and confinement in poor ventilated places. The problem of rashes was mainly because of the exchange of clothes and beddings in the hostels and the communal use of bathing towels such as sacks (improvised towels) and bathing soap.

*“There are patches of malaria, as Mazabuka is mosquito infested because of the sugar plantation”* (Key informant interview)

*“As for me when I came here, after three days, I was surprised to find that I had a lot of rashes over my neck and body. I think even exchanging bathing items, when your friends use it and then you also use it also causes rashes ”* (Juvenile FGD)

#### Erratic funding towards health services

There was also lack of financial resources on the part of the institution as there was no budget line for health care needs assessment. Key informants explained that there was need to provide for the separate budget for health care needs for juveniles if their needs were to be adequately addressed.

*“The funding, I think the separate budget for health needs assessment  for juveniles is required because there are so many needs that should be addressed under health care”*( Key informant interview ).

## Discussion

This study explored the main health needs among juveniles at the Nakambala Correctional institution using an ethics framework. The study showed that most of the juveniles experienced numerous health problems. The common health problems among juveniles were STIs HIV/AIDS, Syphilis, Gonorrhea, flu and colds, abdominal pains, diarrhoea, rashes, Malaria as well as T.B. These findings were similar with the results from a previous study conducted by the American Academy of Pediatrics [1] which showed that globally over 1 million children in conflict with the law in detention and juveniles detained or confined in correctional care facilities have numerous health problems. In the case of STIs, the findings were in line with a needs assessment conducted by Rani et al. [18] in the United States of America where more infections were reported in juvenile correctional institutions than in those children in a general high school population. This high prevalence of the infectious diseases exposes many adolescents to risks they would not ordinarily or freely put themselves in [19]. Such risky situations raises several beneficence and justice concerns and the extent to which national public health policy and decision makers prioritise human rights of adolescents in correctional institutions [19–22]. Talakvadze et al. [20] states that the role of the medical service in the prison is not limited to medical treatment of patients, it also involves provision of social care and preventive activities. Special care should be given to hygiene, prevention of communicable diseases and suicide, elimination of violence, maintaining of social and family relationships.

The findings of the study also showed that the referral systems were non – existent due to lack of coordination between the institution and the nearest health facility. Furthermore, the involvement of untrained personnel in conducting referrals created difficulties in the way referral system were supposed to be administered and undermined adolescents's wellbeing. Effective control measures in the at-risk population need to be implemented to improve retention [21, 22]. The lack of medicine and few heath workers at the health facility where juveniles seek treatment raise additional ethics and health systems challenges. These inadequacies affect the realization of the ethics principle of beneficence as beneficence demands fair distribution of health services [23–30]. The weak refer systems means that it is not possible to guarantee juveniles timely access to services when such services are needed.

The non- availability of adolescent friendly sexual and reproductive health services at the nearest health facility is another ethical dilemma. Lack of youth friendly services have been associated with poor uptake of health services in general populations due to fear by adolescents to mix with adults in reproductive health centres because of cultural norms, values and religious beliefs which tend to restrict sexuality issues to adults [31–36]. Lack of adolescent services present even a more complex scenario especially among the adolescents who apart from having to deal with stigmatization associated with age, also face stigmatization of being classified as deviants. Stigmatization affects the ethics principle of autonomy as it undermines the ability or agency by juveniles to freely choose to seek the health [33–35].

The lack of comprehensive records regarding health care provision to the juveniles both at the institution and at the clinic, health care needs, as well as the inadequate finances have implications on procedural justice [33, 34]. Procedural justice is affected because the lack of appropriate information makes it difficult to come up with fair priorities, or priorities that adequately meet the health needs of the juveniles. In addition, a lack of budget dedicated towards the health needs of the juveniles means that the administration cannot effectively and appropriately support the health needs of the adolescents [30]. The lack of adequate records has also been supported by other studies in Zambia [22, 9], which showed that one of the problems affecting correctional institutions for juveniles was the lack of proper coordination between different government departments that were supposed to provide services to these correctional institutions. Non-availability of documentation created difficulties in the administration of health care services and hindered the steps to improve health care delivery and meeting the heath needs of the juveniles.

Adopting an ethics analysis is essential in identifying and reducing health challenges or gaps among juveniles in that just like the ethics principles, the health care needs or gaps that are associated with the ethics principles are interconnected. For example, health gaps such as inadequate medical and drug supplies as well as non-availability of adolescent friendly health services did not only affect social justice and beneficence principles but also threatened the ethics principle of autonomy by undermining  juveniles’ privacy, liberty and confidentiality with regard to accessing health services. Thus, identification of such ethical dilemmas can help in developing strategies that are not only morally sound by promoting autonomy, beneficence and justice principles in meeting health needs but may also simultaneously trigger a comprehensive approach to health care delivery among juveniles.

### Strengths and limitations of the study

The use of different data collection methods enhanced triangulation of the data which helped in developing an account that is rich and comprehensive [29]. Whereas through the processes of thoroughly documenting the research process, transcribing and reviewing all interviews, we enhanced credibility of the findings [30]. Conducting the study at one institution limited the extent to which the results could be generalised to other institutions. However, considering that the study was conducted at the biggest correctional institution in Zambia, we believe that our results can still provide useful information which can guide stakeholders in addressing the ethics issues associated with the health needs among the juveniles not only in Zambia but also in similar settings.

## Conclusion

There were numerous health problems among the juveniles. Common health problems were sexually transmitted infections, flu and colds, diarrhea, rashes, malaria and poor hygiene. Lack of comprehensive health needs assessment contributed to limited availability of health services which posed threats or ethics concerns related to autonomy, beneficence and social justice among the juveniles. Challenges included weak referral systems due to lack of coordination between the correctional institution and the nearest health facility. There were also inadequate medicines and few heath workers which raised ethical challenges regarding beneficence or the extent to which juveniles benefitted from health services. The non- availability of friendly youth services for sexual and reproductive health problems at the nearest health facility, was another ethical dilemma which constrained health seeking by reducing privacy and thereby undermining juveniles’ autonomy. Furthermore, lack of comprehensive records as well as inadequate finances negatively affected procedural justice as it was difficult to develop and implement fair priorities. Effectively identifying and reducing health challenges among juveniles would require adopting an ethics approach, as it may guide development of strategies that fairly address issues related to juveniles’ liberty to freely seek health services as well as minimise any possible health risks or harm with regard to seeking health services. Improved health service options for adolescents and reduction in health risks may result into enhanced social justice or fairness within the correctional and health institutions. Thus, effectively addressing the health needs and moral dilemmas associated with health needs among adolescents may require paying attention to all the ethics principles in the design and implementation of health care strategies as the ethics principles, just like the health needs or gaps associated with the principles, are intertwined.

## Abbreviations

MCDSS: Ministry of Community Development and Social Services
MOU: Memorandum of Understanding
STIs: Sexually Transmitted Infections
